# Three-dimensional magnetic resonance imaging ultrashort echo-time cones for assessing lung density in pediatric patients

**DOI:** 10.1007/s00247-020-04791-1

**Published:** 2020-08-29

**Authors:** Konstantinos G. Zeimpekis, Julia Geiger, Florian Wiesinger, Gaspar Delso, Christian J. Kellenberger

**Affiliations:** 1 Department of Nuclear Medicine, University Hospital Zurich, Raemistrasse 100, 8091 Zurich, Switzerland; 2 Department of Information Technology and Electrical Engineering, ETH, Zürich, Switzerland; 3 Department of Diagnostic Imaging, University Children’s Hospital Zürich, Zürich, Switzerland; 4 Children’s Research Center, University Children’s Hospital Zürich, Zürich, Switzerland; 5 GE Healthcare, Waukesha, WI USA

**Keywords:** Adolescents, Children, Cones, Cystic fibrosis, Lung density, Magnetic resonance imaging, Ultra-short echo time

## Abstract

**Background:**

MRI of lung parenchyma is challenging because of the rapid decay of signal by susceptibility effects of aerated lung on routine fast spin-echo sequences.

**Objective:**

To assess lung signal intensity in children on ultrashort echo-time sequences in comparison to a fast spin-echo technique.

**Materials and methods:**

We conducted a retrospective study of lung MRI obtained in 30 patients (median age 5 years, range 2 months to 18 years) including 15 with normal lungs and 15 with cystic fibrosis. On a fast spin-echo sequence with radial readout and an ultrashort echo-time sequence, both lungs were segmented and signal intensities were extracted. We compared lung-to-background signal ratios and histogram analysis between the two patient cohorts using non-parametric tests and correlation analysis.

**Results:**

On ultrashort echo-time the lung-to-background ratio was age-dependent, ranging from 3.15 to 1.33 with high negative correlation (R_s_ = −0.86). Signal in posterior dependent portions of the lung was 18% and 11% higher than that of the anterior lung for age groups 0–2 and 2–18 years, respectively. The fast spin-echo sequence showed no variation of signal ratios by age or location, with a median of 0.99 (0.98–1.02). Histograms of ultrashort echo-time slices between controls and children with aggravated cystic fibrosis with mucus plugging and wall thickening exhibited significant discrepancies that differentiated between normal and pathological lungs.

**Conclusion:**

Signal intensity of lung on ultrashort echo-time is higher than that on fast spin-echo sequences, is age-dependent and shows a gravity-dependent anterior to posterior gradient. This signal variation appears similar to lung density described on CT.

## Introduction

Computed tomography (CT) is considered the reference standard for imaging the lung parenchyma. Nonetheless MRI is being used increasingly for assessing the lung and airways in children [1–4]. Newer MRI techniques such as ultrashort echo-time and zero echo-time sequences have recently boosted the clinical MRI application to lung morphology and pathology [5–10]. The big advantage of MRI for lung imaging in children would be that it does not involve ionizing radiation — which poses a substantial risk, especially in young children, who are the most sensitive [11] — thus MRI could be used as radiation-free alternative to follow-up CT scans in pediatric patients [12]. This study involved children and adolescents with cystic fibrosis, with the goal of validating the diagnostic value of our ultrashort echo-time sequence. Other patients might benefit from dose mitigation.

The proton density of the lung is 10 times lower than that of other organs, and the T2* relaxation time for the lung parenchyma is similarly short (1.43±0.41 ms at 1.5 tesla [T] [13] and 0.85±0.1 ms at 3 T [14]). In addition to the low proton density, strong susceptibility effects from air–tissue interface (alveolar microstructure) contribute to the fast signal decay. Conventional protocols using spin-echo or gradient-echo pulse sequences with echo times in the range of milliseconds cannot detect the lung signal because it has already been decayed before it can be acquired.

Three-dimensional cones (GE Healthcare, Waukesha, WI) is an ultrashort echo-time sequence with a twisting radial k-space trajectory and is a member of non-Cartesian acquisition schemes in MRI. Cones can achieve echo times as short as 30 μs [15]; thus, it can deliver images that capture rapid decaying signal from lung parenchyma. It includes an extended alternative version to 3-D projection reconstruction, where the spokes twist around one of the axes, resulting in shorter scan times with increased signal-to-noise ratio. The more twisting is added to the spokes, the more the readout of each spoke is prolonged, leading to the coverage of k-space being done in a more efficient and faster way, achieving overall shorter scan times (on average 2 times shorter) because fewer spokes are required to achieve the same k-space filling [15]. The whole k-space is acquired through many size-varying cones, each of them made of a different number of spokes.

Recent studies [16–18] have shown ultrashort echo-time performance for pediatric cystic fibrosis lung cases, but to the best of our knowledge they were limited to diagnostics, while others performed lung density measurements related to CT [19–22]. These studies did not test lung density visualization depending on age with 3-D ultrashort echo-time cones.

The aim of this study was to investigate the performance of ultrashort echo time for depicting lung signal intensities in relation to patients’ age and compare it against the routinely used T2-weighted fast spin-echo sequence. Our hypothesis was that ultrashort echo-time might have higher diagnostic value compared to the reference sequence as a result of its higher lung-to-background signal intensity ratio (lung-to-background ratio) and that the lung signal intensity could be correlated to lung density comparable to CT.

## Materials and methods

### Study sample

This retrospective study included 30 children and adolescents who underwent lung MRI at 1.5 T at a tertiary university children’s hospital between 2015 and 2019. Patients were split into two groups: 15 patients (10 females, 5 males, median age 6 years, range 0–18 years) with morphologically normal lungs were used as a control group and 15 patients (4 females, 11 males, median age 4 years, range 1–17 years) were diagnosed with cystic fibrosis. Indications for chest MRI examinations in the control group were as follows: 13 patients had respiratory symptoms (wheezing, dyspnea, airway compression, cough, recurrent pneumonia) and were examined to rule out vascular or pulmonary malformation or mediastinal tumor, from which 3 patients were found to have innominate artery compression syndrome. One patient was referred to rule out metastases in ovarian germ cell tumor in combination with whole-body MRI, while another had immunodeficiency. The included patients and their parents gave consent to retrospective data analysis. The study was approved by the responsible government ethics committee.

### Imaging techniques

All patients were scanned according to our institutional lung protocol [4], which has included an axial 3-D ultrashort echo-time cones sequence since 2015. The patients were scanned in supine position on a 1.5-T system (Discovery MR450; GE Healthcare, Waukesha, WI). The MRI protocol included several sequences for dedicated lung imaging such as a respiratory-gated T2-weighted fast spin-echo sequence with periodically rotated overlapping parallel lines with enhanced reconstruction (PROPELLER) in transverse orientation, which we used as a reference sequence for the respiratory-gated cones sequence.

Respiratory gating was achieved by having patients wear belts to capture the physiological diaphragmatic signal. Cones sequence was twice as fast as the fast spin-echo sequence. Twenty-four patients (9 controls/15 with cystic fibrosis) were scanned with a surface coil (32-channel cardiac, GE Healthcare) while 6 patients were scanned with a flex surface coil (12 channels, GEM Flex Suite, GE Healthcare). Seven controls and nine children with cystic fibrosis were sedated during the scans. The acquisition parameters for both sequences are shown in Table 1.

**Table 1:** Acquisition parameters of cones and fast spin echo

Parameter	Cones	Fast spin echo
Repetition time, ms	3.7	12,000
Echo time, ms	0.028	72
Spoke readout time, ms	740	–
Flip angle	5°	140°
Field of view, cm^2^	26×26 or 36×36	26×26 or 36×36
Slice thickness, mm	2.0 or 3.0	4.0–8.0
In-plane resolution, mm	1.3	0.8
Acquisition matrix	384×384	288×288
Receiver bandwidth, kHz	256	62.5
Acquisition time	3–5 min	~6 min

### Quantitative analysis

**Fig. 1:**
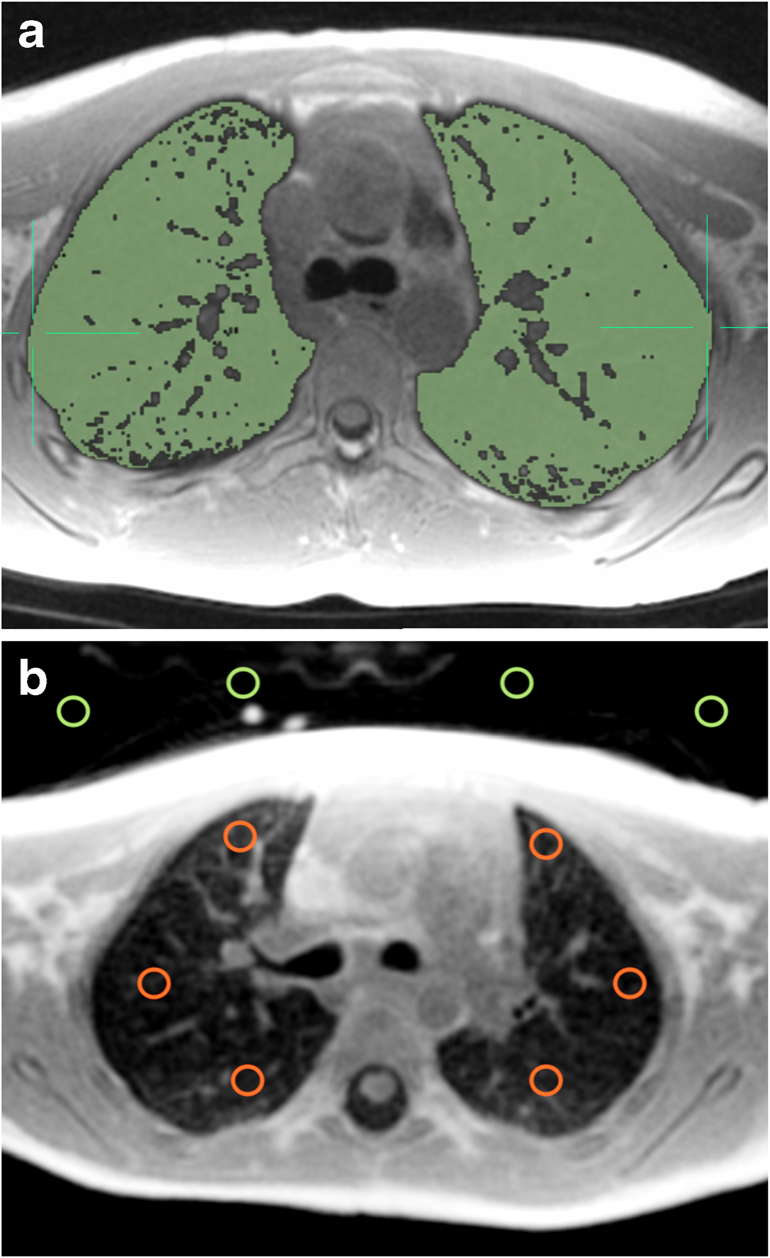
Manual lung segmentation by thresholding and region-of-interest placement in a 4-year-old boy. **a** Axial slice shows the manual segmentation of lung parenchyma (*green*). **b** Axial slice with background (*green circles*) and lung (*red circles*) regions-of-interest

Signal intensity of both lungs and background were measured for both cohorts in both sequences. To measure the lung signal intensity, manual segmentation of both lungs was applied by appropriate intensity thresholding, excluding high-intensity vessels (Fig. 1). Lung segmentation was performed using the open-source image analysis software OsiriX MD version 11.03 (Pixmeo Sarl 2016, Bernex, Switzerland). To measure the background signal intensity, we drew four two-dimensional regions-of-interest, each with size of 20 mm^2^, in the air anterior to the thorax in each slice (Fig. 1). We calculated the average of all region-of-interest signal intensities. One additional two-dimensional region-of-interest of variable size was drawn inside the trachea on an axial slice at the carina level to measure the signal intensity of the air column within the trachea.

For controls, two-dimensional regions-of-interest with a size of 20 mm^2^ were drawn in anterior, middle and posterior parts of both lungs (three regions-of-interest per lung), for both sequences at the level of the carina (Fig. 1). The regions-of-interest were placed carefully in lung parenchyma areas without including vessels to avoid signal contamination.

The whole-lung signal intensity was calculated as the average signal intensity of both segmented lungs. For the different lung anatomical regions for controls, we calculated average anterior (respectively middle and posterior) signal intensity as the signal average of the anterior regions-of-interest in left and right lungs (respectively, middle and posterior regions-of-interest).

The lung-to-background ratio for the whole lung was calculated as the average lung-to-background signal intensity.

For signal-to-noise ratio and contrast-to-noise ratio, the region-of-interest in the trachea was taken as the reference background signal because it is more representative for the noise level within the lungs. The signal-to-noise ratio was calculated as the ratio of the lung signal intensity to the standard deviation of the signal intensity of the trachea’s region-of-interest. The contrast-to-noise ratio was defined as the ratio of the difference of the lung and trachea signal instensities to the standard deviation of signal intensity of the trachea’s region-of-interest.

Histograms of a cones axial slice for controls and for two children with cystic fibrosis, one with mucus plugging and the second with wall thickening, were extracted, using the pyOsiriX tool [23] to measure skewness and kurtosis of the pixel intensity distributions. Skewness describes the asymmetry of the distribution, while kurtosis shows the range of the intensity distribution.

### Statistical analysis

We used descriptive statistics to display the lung-to-background ratio in terms of mean and median with the corresponding standard deviation. Two-tailed Wilcoxon signed rank test and Mann–Whitney *U* test were used to assess whether the difference of the lung-to-background ratio between sequences and patient groups were statistically significant (*P-*values ≤0.05). Spearman rank correlation test was performed to test dependency of the lung-to-background ratio to age. For the lung-to-background ratio comparison between controls and cystic fibrosis patients, only children older than 2 years were considered because no newborns with cystic fibrosis were included in our study; therefore, the sample population consisted of 11 controls and 13 children with cystic fibrosis. Statistical analysis was performed in R (version 3.5.1; R Foundation for Statistical Computing, Vienna, Austria).

## Results

**Fig. 2:**
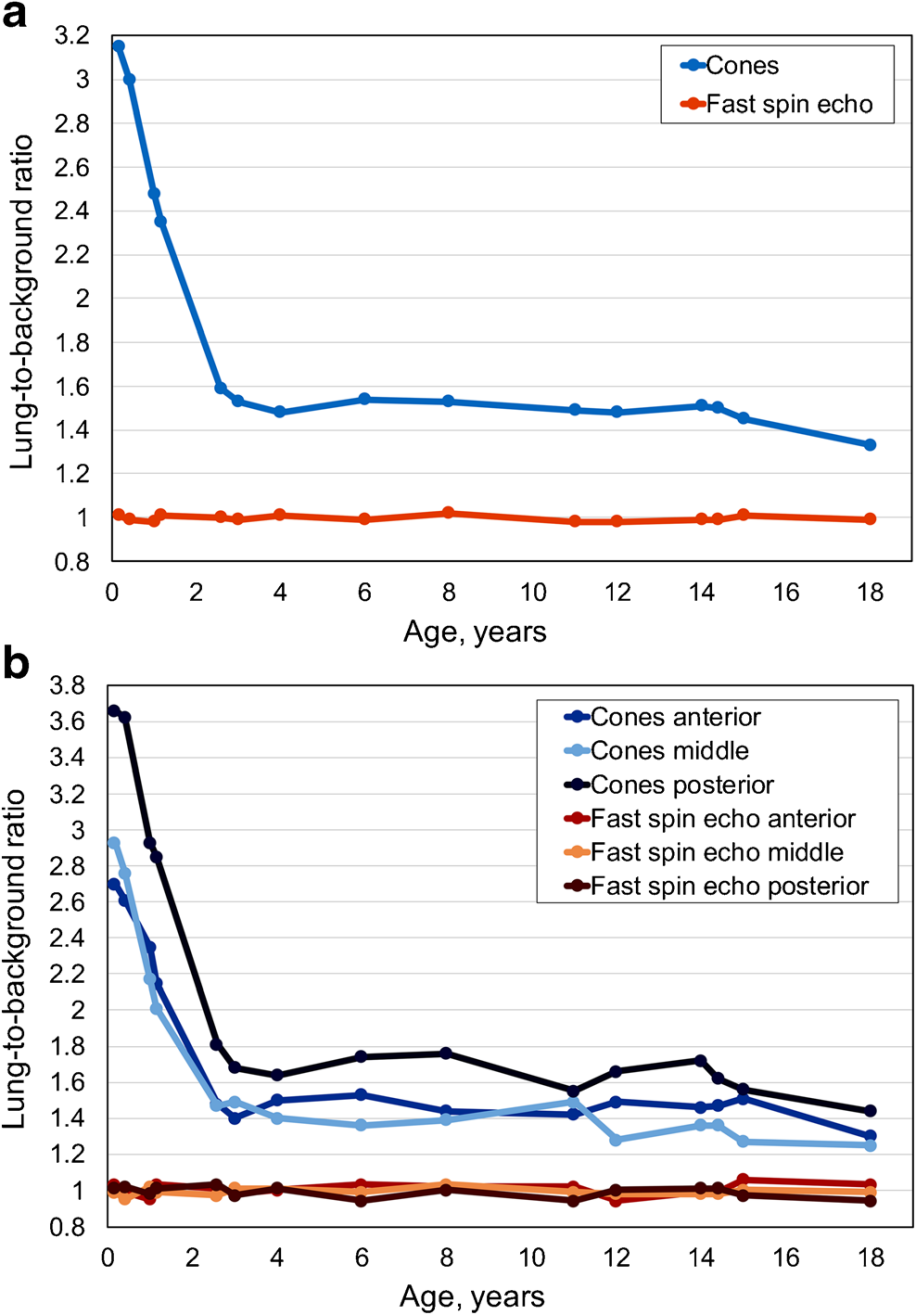
Lung-to-background signal intensity ratio curve depending on age and gravity gradient. **a** Whole**-**lung-to-background ratio curve for cones and fast spin-echo in controls related to age. **b** Regional lung-to-background ratio for anterior, middle and posterior areas for cones and fast spin-echo in controls related to age

For the 15 controls, the lung-to-background ratio for cones was higher than 1 for all ages (Fig. 2). The highest lung-to-background ratio was exhibited for newborns with values of 3.15 and 3.00 for the patients ages 2 months and 6 months, respectively. For the patients ages 12 months and 14 months we depicted a lung-to-background ratio drop to 2.48 and 2.35, respectively. The lung-to-background ratio was almost stable between the ages of 2 years and 18 years (1.60 to 1.33). There was a high negative correlation between the lung-to-background ratio of cones and increasing age (Spearman correlation coefficient R_s_ = −0.86; *P*<0.001).

For the fast spin-echo sequence, the lung-to-background ratio was approximately 1 for all ages, with negligible variations. In cases with lung-to-background ratio less than 1, the background signal was slightly higher than the lung signal from noise variations. Qualitatively, cones showed higher contrast inside the lung compared to our reference sequence. The difference of the lung-to-background ratio between cones and fast spin echo was found to be statistically significant (*P*<0.002).

The regional age-dependent lung-to-background ratio curves for the three defined regions-of-interest in the anterior, middle and posterior pulmonary regions are shown in Fig. 2. The posterior lung-to-background ratio was increased compared to the anterior and middle lung-to-background ratios for all ages and was highest for newborns, with a decrease until the age of 2 years. There were no differences in lung-to-background ratio in the fast spin-echo sequence with regard to the region-of-interest location.

**Fig. 3:**
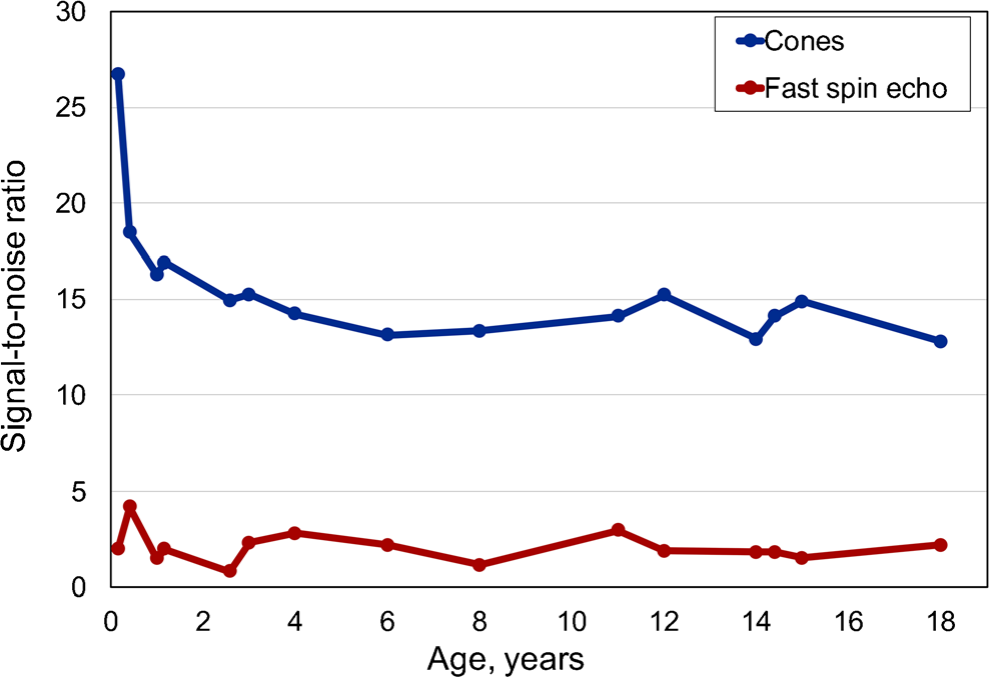
Whole lung signal-to-noise ratio curves for cones and fast spin echo in controls related to age

**Fig. 4:**
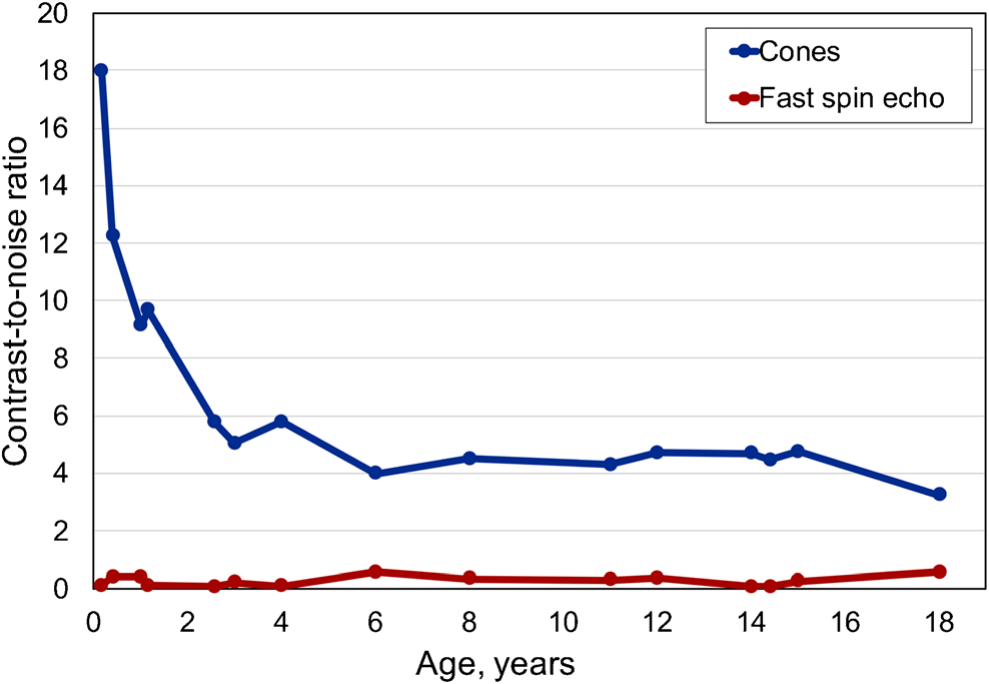
Whole-lung-to-trachea contrast-to-noise ratio curves for cones and fast spin echo in controls related to age

Signal-to-noise ratio and contrast-to-noise ratio curves (Figs. 3 and 4) were higher for cones than for the fast spin-echo sequence for all ages. We observed the highest values in newborns and a rapid decrease to the age of 2 years. From newborns to young adults, signal-to-noise ratio ranged from 27 to 14, while contrast-to-noise ratio dropped from 18.0 to 3.6. Signal-to-noise ratio of the fast spin-echo sequence was 0.81 in newborns to 4.20 in young adults, while contrast-to-noise ratio was 0.06 to 0.40.

**Fig. 5:**
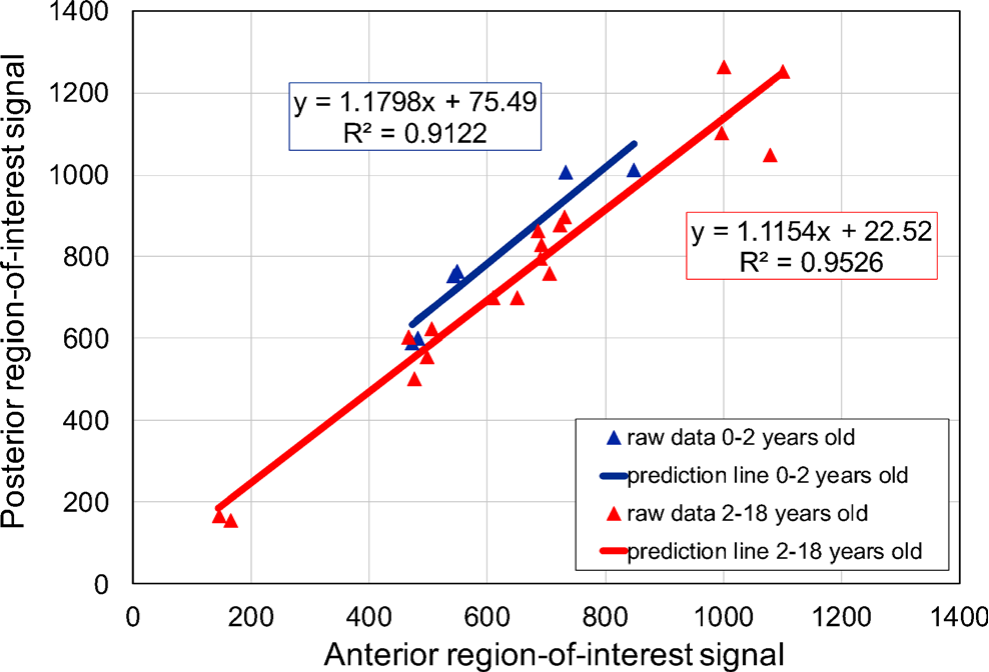
Correlation of anterior to posterior region-of-interest signal intensities for cones in the control age groups of 0–2 years and 2–18 years

We divided the control group into two age groups (0–2 years and 2–18 years) to show the relationship between anterior and posterior lung-to-background ratio. Figure 5 shows the correlation of anterior (x-axis) and posterior (y-axis) regions-of-interest for both age groups. The corresponding correlation coefficients were R_1_^2^=0.912 and R_2_^2^=0.953, which showed high correlation of over 91% and 95%, respectively, for the two age groups. In addition, the posterior lung-to-background ratio was 18% higher than the anterior lung-to-background ratio in the younger age group and 12% higher in the older age group.

For the cystic fibrosis cohort, a variety of pathologies was found, like bronchiectasis, bronchial wall thickening, mucus plugging and air trapping, depending on the stage of the disease. The median lung-to-background ratio was not significantly increased (*P*>0.2) between cystic fibrosis patients and controls for all ages (1.75–1.53).

Mild cystic fibrosis cases were the most common; the remaining cystic fibrosis patients had predominantly mucus plugging and wall thickening rather than air trapping. Minimum and maximum lung-to-background ratio values for controls and children with cystic fibrosis were 1.33–1.47 and 3.15–2.12, respectively.

**Fig. 6:**
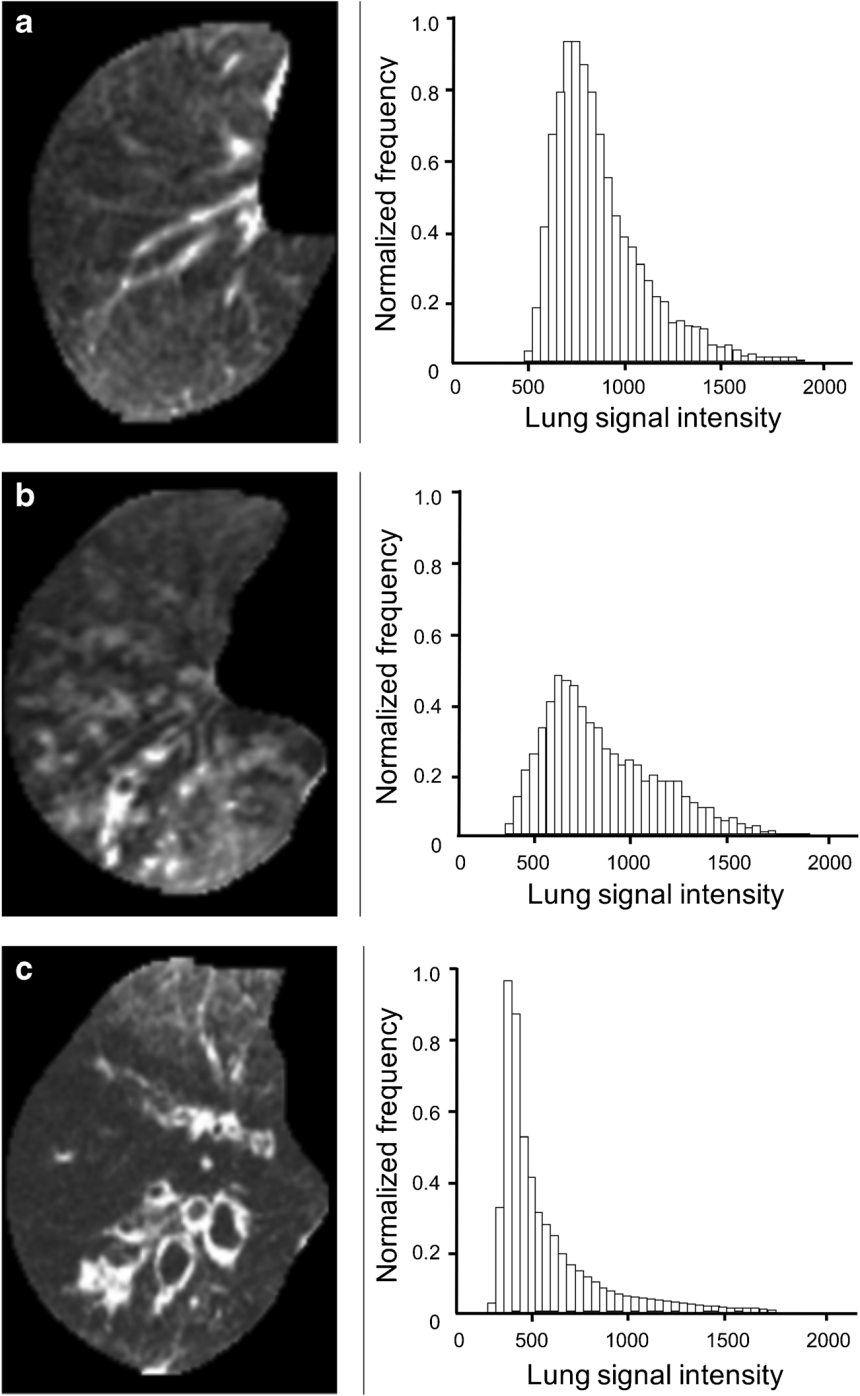
Histograms of pixel intensities of the segmented right lung comparing an 8-year-old male control patient (**a**), an 11-year-old girl with cystic fibrosis with mucus plugging (**b**) and a 14-year-old girl with cystic fibrosis with wall thickening (**c**) scanned with cones

We selected only the right lung for the histogram analysis. We assessed all controls and two children with cystic fibrosis with prominent mucus plugging and wall thickening. Only the aggravated cases were taken into consideration because mild cystic fibrosis cases didn’t present large pixel distribution changes to the controls. Figure 6 shows a representative axial slice of the segmented lung of a control subject and of both children with cystic fibrosis. All histograms were positively skewed. The average skewness among all controls was 1.75; for the child with cystic fibrosis and mucus plugging, 0.87; and for the child with wall thickening, 1.90; the kurtosis values were 1.6, 0.5 and 4.4, respectively.

**Fig. 7:**
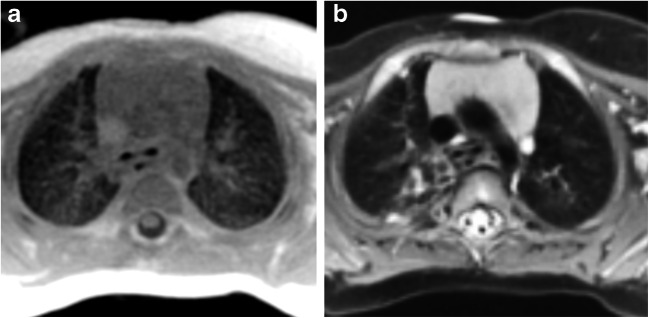
Axial slices at carina level of a 5-month-old male control scanned with (**a**) cones and (**b**) fast spin echo. Cones provides higher contrast inside the lungs compared to the background and the signal of the trachea, which indicates capture of the lung parenchyma. Fast spin echo does not show any higher lung contrast. Qualitatively, cones delivers the highest lung contrast for newborns, compared to older patients (shown in Figs. 8 and 9)

**Fig. 8:**
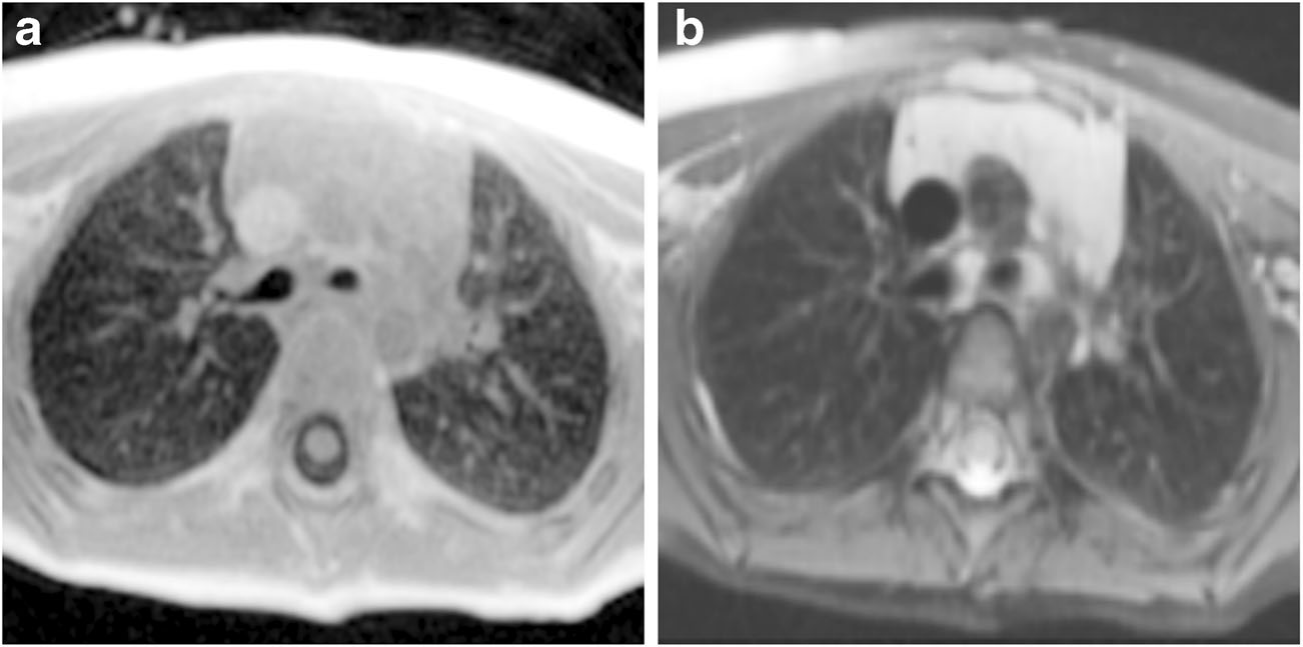
Axial slices at the carina level of a 31-month-old girl control subject scanned with (**a**) cones and (**b**) fast spin-echo sequence. Cones provides higher contrast inside the lungs compared to the background and the signal of the trachea, which indicates capture of lung parenchyma. Fast spin-echo sequence does not show any higher lung contrast

**Fig. 9:**
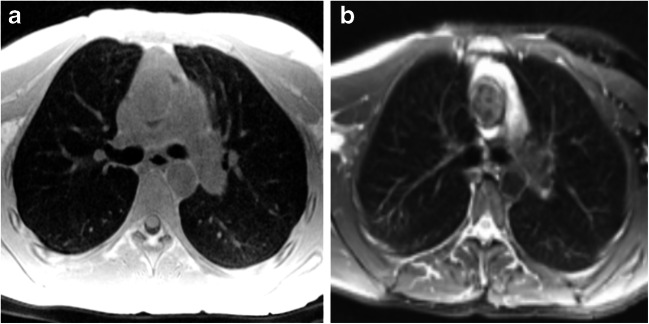
Axial slices at carina level of an 18-year-old female control subject scanned with (**a**) cones and (**b**) fast spin echo sequence. Using cones provides higher contrast inside the lungs compared to the background and the signal of the trachea, which indicates capture of lung parenchyma. Fast spin echo does not show any higher lung contrast. Compared to the younger patients (shown in Figs. 7 and 8), cones provides the smallest lung contrast that is associated with lower lung signal intensity with increasing age

The qualitative assessment of cones and the fast spin-echo sequence revealed increased lung parenchymal intensity in young children depicted by cones that decreased with increasing age (Figs. 7, 8 and 9). The fast spin-echo sequence failed to show any contrast change depending on age.

## Discussion

With this retrospective study in children who underwent clinically indicated lung MRI, we showed that lung signal intensity measured by ultrashort echo-time cones correlates to lung parenchymal density while a standard fast spin-echo sequence failed to show age-dependent signal changes.

While T2* is very short in the lungs, T2 is still relatively long [24]. The fast spin-echo sequence has a radial acquisition scheme wherein a group of parallel data lines is acquired together while rotating around the center of k-space, leading to inherent oversampling of the central k-space [25]. It is more robust to motion, providing in-plane motion correction along with rotation and translation [26, 27], and provides better image quality [28] and is widely used in the MRI of the lung [29–31]. Now it is being used with respiratory gating for children who are unable to make breath-hold scans, for example children with cystic fibrosis [32].

The age-dependency of the lung signal intensity from newborns up to 2 years based on cones imaging was in agreement with the CT findings in the literature. The lung density was higher for newborns and infants until the age of 2 and then stabilized until the age of 18 years with a steady but mild decrease [33], while the lung-to-background ratio profile followed the density trend of the CT findings. Newborns had three times higher lung signal intensity than the background. As from the age of 2 years until adulthood, the lung-to-background ratio remained stable at about 1.5.

Another finding of the study was the demonstration of the vertical gravity gradient of the lung density. Posterior lung-to-background ratio (as well as posterior signal-to-noise and contrast-to-noise ratios) was constantly higher than the anterior lung-to-background ratio (respectively anterior signal-to-noise and contrast-to-noise ratios) for all patients independent of age, as previously shown in the literature [34–37]. In addition, the lung-to-background ratio of the middle lung part appeared to be lower than the anterior lung-to-background ratio, agreeing with previous findings [38] that showed that the average density of the lung region near the thoracic wall was lower than the average density of the whole lung because the middle region-of-interest was placed near the thoracic wall. The linear regression fit for the two age groups, newborns to 2 years and older than 2 years, showed that the posterior intensity was higher than the anterior intensity.

Cones detected higher lung signal intensity, showing lung parenchyma for all ages compared to the reference sequence. The fast spin-echo sequence was not capable of detecting the parenchyma because its echo time was substantially higher than that of the lung T2* at 1.5 T. Fast spin-echo sequences are not suited to assess lung parenchyma of aerated lung, although they are able to depict consolidations. Whole-lung signal-to-noise and contrast-to-noise ratios of cones were increased compared to our reference sequence for all patients.

In accordance with previous studies, the comparison of lung-to-background ratio of controls versus people with cystic fibrosis did not reveal a statistically significant difference [39]. However, the statistics showed that the median lung-to-background ratio of the children with cystic fibrosis was higher than that of the controls, while the whole interquartile range of the lung-to-background ratio of the children with cystic fibrosis was narrower and was within that of the control patients. This could further show that there were both increases and decreases in regional lung intensity in the presence of cystic fibrosis pathology, from either air trapping or infiltrative changes like wall thickening and mucus plugging, as previously suggested [40]. The higher median lung-to-background ratio might be attributed to more cases with mucus retention that increases the regional lung intensity.

Analysis of the pixel-intensity histograms of the right lung of the control patients and the child with cystic fibrosis with prominent mucus plugging showed different profiles with variations in skewness and kurtosis that were in agreement with the CT findings by de Lavernhe et al. [40]. Skewness and kurtosis of the child with cystic fibrosis with mucus plugging were significantly lower than those of the controls. These decreases in skewness and kurtosis corresponded to the increase of the scattering and reduction of the asymmetry of the density distribution from lung heterogeneity within the same slice. Using this approach, cones revealed another quantitative aspect of its use for children with cystic fibrosis. In contrast, the skewness and the kurtosis of the child with cystic fibrosis with wall thickening were higher compared to those of the controls. Cones could be further used for differentiating among cystic fibrosis cases. Qualitatively, the histograms exhibited different distribution profiles.

We can estimate parenchymal lung density with cones but not with a fast spin-echo sequence, both in normal lungs and in lungs with pathology, because lung intensities measured on cones show the same age and gravity dependence as lung density measured on CT. The possibility to quantify parenchymal lung intensity (as a measure of lung density) by MRI opens new possibilities for grading or assessing lung pathology, particularly diffuse pathology such as overinflation (in congenital lung lesions or in small airways disease, e.g., cystic fibrosis), lung hypoplasia (in children with congenital diaphragmatic hernia) or emphysema.

This study has some limitations. MRI signal depends on parameters set by the operator as well as patient characteristics. Background and lung signal for that matter vary even between patients of the same age. Background signal distribution varies along field-of-view as well, with the noise level being the lowest at the top of the coronal field-of-view near the neck and highest at the bottom over the abdomen. The noise distribution was not homogeneous, so noise appeared mild in the upper part of the body near the neck and more prominent toward the abdomen. To overcome noise inhomogeneities, we used the average as a reliable metric of noise over the whole covered area.

Patient size and weight affect the signal intensity as well. Optimal coil configuration to cover the prescribed field-of-view sufficiently seems to deliver the lowest noise level and there is a variability of the coil setup among different children. Another limitation is that the children were not scanned with the same coil (most of them were scanned with the 32-channel cardiac coil but some were scanned with a full flex coil).

The slice thickness of cones varied between 2 mm and 3 mm, whereas the in-plane resolution was fixed. However, calculating the lung-to-background ratio was expected to cancel these intrapatient signal differences caused by different voxel sizes. The fast spin-echo scans were acquired with thicker slices than cones but with higher resolution, leading to smaller voxels compared to cones. Cones had 3.4–5.0 mm^3^ while the reference sequence was acquired with 2.6–5.1 mm^3^. Thus, in some cases cones had larger voxels while in most cases the intersequence voxel size was comparable. The MRI signal is proportional to the voxel size; however, no image acquired with the fast spin echo, regardless of voxel size, could provide any higher lung signal.

The regions-of-interest for assessing the gravity gradient were not drawn at the exact same positions in all children because of their different sizes but were definitely at the same anatomical regions. Insufficient coil intensity correction might have contributed to the middle lung region-of-interest lung-to-background ratio being lower than the anterior one. Some of the newborns presented with atelectasis from sedation. In these cases, regions-of-interest had to be placed next to the atelectasis to avoid errors in signal intensity. Because the whole lung segmentation was done by appropriate thresholding, atelectatic areas were not included in the measurement of the signal intensity.

Because the scans were acquired with respiratory gating, the lung signal came predominantly from the end-expiration phase. However, some of the inhaled air (except the residual lung volume) might have been present in some cases because of irregular breathing patterns, leading to signal loss and potentially negligible or mild motion artifacts. The small sample is another limitation that should be considered, and differences between female and male patients were ignored even though female lung density has been shown to be higher than male [38]. Despite that, we were able to show the age dependence of the signal intensity of the lung. The effect of sedation on the extraction of the signal intensities of lung volumes was deemed negligible because patients of all ages were sedated and the decrease in older patients was still evident.

Finally, we did not compare MRI lung signal intensity with CT lung density in the same children because of the retrospective study type. Lung is among the most radiosensitive organs and we avoided unnecessary exposure from CT. Although this is not a hard quantification, the age-dependent curve of the lung signal intensities by cones follows the same fashion as lung density in CT literature.

## Conclusion

The lung signal intensity assessed by cones seems to represent lung density. This might open new possibilities for MR quantification of lung pathologies.
